# Supplementary material for: Covalent organic framework nanofluidic membrane as a platform for highly sensitive bionic thermosensation
Source: Nat Commun. 2021 Mar 23;12:1844. doi: 10.1038/s41467-021-22141-z (PMC7988099; doi:10.1038/s41467-021-22141-z)
Supplement: Supplementary file 1 — Supplementary Information [file 41467_2021_22141_MOESM1_ESM.pdf]

Supplementary Information

Covalent Organic Framework Nanofluidic Membrane as a Platform for Highly Sensitive Bionic Thermosensation

Pengcheng Zhang,[1] Sifan Chen,[1] Changjia Zhu,[1,2] Linxiao Hou,[1] Weipeng Xian,[1] Xiuhui Zuo,[1] Qinghua Zhang,[1] Lin Zhang,[1] Shengqian Ma,[2,]* Qi. Sun,[1,]*

[1]Zhejiang Provincial Key Laboratory of Advanced Chemical Engineering Manufacture Technology, College of Chemical and Biological Engineering, Zhejiang University, Hangzhou, 310027, China; [2]Department of Chemistry, University of North Texas, 1508 W Mulberry St Denton, TX 76201, United States.

*Correspondence to: sunqichs@zju.edu.cn; shengqian.ma@unt.edu

Materials synthesis

Fabrication of TpTag-COF

TpTag-COF powder was synthesized *via* the condensation of Tp (42 mg, 0.2 mmol) and Tag (28 mg, 0.2 mmol) in a sealed Pyrex tube. The mixtures (2.6 mL, dioxane/water=1/0.3) were charged into the Pyrex tube and sonicated for 20 min. The mixtures were degassed under liquid N_2 (77 K) by three freeze-pump-thaw cycles. The Pyrex tube was then vacuum-sealed and held at room temperature until the reaction mixtures attended the room temperature. The sealed Pyrex tube containing the reaction mixture was kept at 120 °C for 3 days. The TpTag-COF was obtained as a brown powder after being washed thoroughly with water and acetone in sequence and then dried under vacuum.

Fabrication of TpTag-COF/PAN

The COF active layer was formed *via* interface polymerization on the surface of a PAN ultrafiltration membrane. The PAN support was vertically placed in the middle of a homemade diffusion cell, resulting in each volume of 7 cm^3 (see Figure S1). An aqueous solution of Tag (9.9 mg, 0.090 mmol) dissolved in 3 M acetic acid (7 mL) and the organic phase with Tp (14.7 mg, 0.070 mmol) dissolved in the mixture of ethyl acetate/mesitylene (V/V=1/5, 7 mL) were simultaneously introduced into the two sides of the diffusion cell using two pipettes. The reaction mixture was kept at room temperature for 3 days. The resulting membrane was rinsed with ethanol, methanol, and water in sequence to remove any residual monomers, catalyst, and organic solvents.

Fabrication of BtTag/PAN

The BtTag active layer was synthesized by interfacial polymerization. 1,3,5-Benzenetricarbonyl trichloride-hexane solution (80 mg in 30 mL) was carefully poured on the surface of Tag aqueous solution (40 mg in 30 mL, pH = 12) at room temperature. After reacting for 60 s, the fabricated free-standing nanofilm was collected from the interface with a pre-submerged strainer mesh with PAN substrate. The resulting membrane was further undergone thermal treatment (80 °C) for 10 min to increase the crosslinking degree. After that, the membrane was compacted at 2 bar for at least 1 h to improve the adhesion between the BtTag active layer and substrate.

Permselectivity evaluation

For investigating the ion transport property of nanochannels, the ion current was recorded by CHI660E with a homemade electrochemical cell. The voltage was scanned with a step of 0.01 V s^{-1} using Ag/AgCl electrodes. The K^+/Cl^- selectivity of TpTag-COF/PAN was evaluated by determining the reversal potentials with different KCl concentration gradients. The X-intercepts (V_r) of the I−V plots represent the average reversal potentials. The permselectivity was determined according to the Goldman-Hodgin-Katz equation. The permselectivity of other electrolytes were evaluated following the same procedures as that of KCl.

Thermoelectric response of TpTag-COF/PAN

The thermoelectric response was recorded by a homemade setup. A micro-ceramic heater (Zhuhai Huiyou Electronics, China) was employed to regulate the solution's temperature. A direct current power (HSPY-60-5, Hanshengpuyuan, China) was connected to the heater to control the heating rate and temperature range. A pair of temperature microsensors (PT100, Tenghui Wenkong Instruments, China) were immersed in two solutions to measure the real-time temperature, which was recorded by a temperature sensor (THMA temperature recorder, Tenghui Wenkong Instruments, China). The measurement accuracy and working range of the temperature sensor are ± 0.1 K and −173.15 − 473.15 K, respectively. The transmembrane potential was synchronously collected by a CHI660E electrochemical workstation using two Ag/AgCl electrodes. The time resolutions of temperature recorder and transmembrane potential are both 1 s.

Theoretical Derivation of Thermoelectric Response

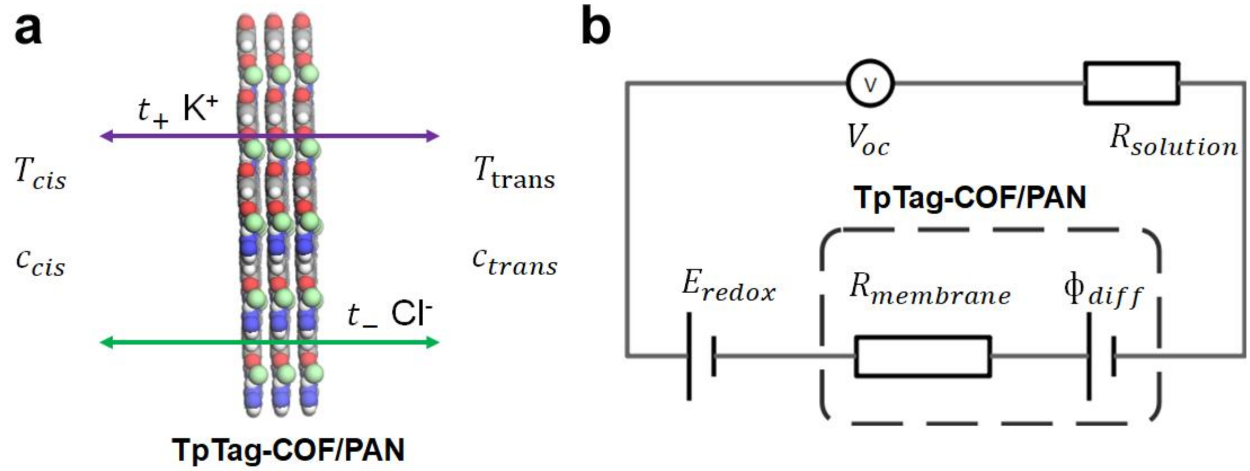

The schematic model shown above is used to derive the theoretical thermoelectric response of TpTag-COF/PAN. Two chambers, designated as *cis* and *trans*, contain systematic electrolyte (KCl) with an activity of a_{cis} and a_{trans}. In this work, KCl was used as the electrolyte. Therefore, the subscripts, + and -, refer to K^+ and Cl^-, respectively. Accordingly, the temperature of the solutions is T_{cis} and T_{trans}. Given a quasi-steady state of ion transport through the membrane from one solution to another, the following equation can be considered,

$$\Delta G = 0 \quad (1)$$

According to the variation of Gibbs free energy of a system (dG),

$$dG = -SdT + Vdp + \sum_{i=1}^{n} \mu_i dn_i \quad (2)$$

Where G, S, T, V, and p are the Gibbs free energy, entropy, temperature, volume, and pressure of the solution. μ_i and n_i are the chemical potential and the chemical amount of species i. Therefore, a temperature change will drive the transport of ions in the opposite direction of the temperature gradient.

In the *cis* or *trans* chamber, the system is held at constant temperature and pressure, dG is presented as follows,

$$dG = \sum_{i=1}^{n} \mu_i dn_i \quad (3)$$

Where n_i is the molar number of species i. $\mu_{i,cis}$ $(j = cis, trans)$ is the chemical potential of ions, which is given by,

$$\mu_{i,j} = \mu_{i,j}^{\ominus} + RT_j ln a_{i,j} + z_i F \phi_j \quad (4)$$

Where $\mu_{i,j}^{\ominus}$, $a_{i,j}$, and z_i are the standard chemical potential, the activity, and the charge valence of i ions in j chamber. R and F are the gas constant and the Faraday constant, respectively. ϕ_j is the inner potential of j chamber. T is the absolute temperature.

Note that the variation of the activity with temperature is neglected here, due to a small temperature change (10 K, maximum) in this work. Considering the electroneutrality condition in two solutions of

KCl, we have,

$$z_+ = 1, \qquad z_- = -1 \tag{5}$$

$$a_{+,cis} = a_{-,cis} = a_{cis}, a_{+,trans} = a_{-,trans} = a_{trans} \tag{6}$$

From above equations, the following one can be derived,

$$\phi_{cis} - \phi_{trans} = \frac{n_+ + n_-}{n_+ - n_-}\frac{R}{F}(T_{trans} ln a_{trans} - T_{cis} ln a_{cis}) \tag{7}$$

Then we can define the transmembrane diffusion potential (ϕ_{diff}) as,

$$\phi_{diff} = \phi_{cis} - \phi_{trans} \tag{8}$$

Ion transport number, also called the transference number, is the fraction of the total electrical current carried in an electrolyte by a given ionic species i,

$$t_i = \frac{I_i}{I_{total}} \tag{9}$$

For a steady flow of charge through a surface, the current I can be calculated with the following equation,

$$I_i = \frac{Q_i}{t} = \frac{z_i e N_A n_i}{t} \tag{10}$$

where Q is the electric charge transferred through the surface over a time t, z_i is the charge valence of ions, e is the elementary charge, N_A is Avogadro constant, and n_i is the molar number of species i.

From above two equations, we can get the following equations,

$$\frac{n_+ + n_-}{n_+ - n_-} = \frac{t_+ - t_-}{t_+ + t_-} = (2t_+ - 1) \tag{11}$$

$$\phi_{diff} = (2t_+ - 1)\frac{R}{F}(T_{trans} ln a_{trans} - T_{cis} ln a_{cis}) \tag{12}$$

According to Supplementary Equation **12,** ϕ_{diff} is a function of both temperature and salt activity in two solutions.

The schematic model **b** shows the equivalent circuit of experimental system, in which the open-circuit potential (V_{oc}) is measured with two silver/silver chloride (Ag/AgCl) electrodes immersed in two solutions. It is the sum of thermoresponsive transmembrane potential (ϕ_{diff}, the direction from *cis* to *trans* is defined to be positive), the difference of redox potentials of two AgCl/Ag electrodes (E_{redox}) and the voltage drops across the membrane ($iR_{membrane}$) and in solution ($iR_{solution}$),

$$V_{oc} = V_{cis} - V_{trans} = -\left(\phi_{diff} + E_{redox} + iR_{membrane} + iR_{solution}\right) \tag{13}$$

where i is the ionic current, $R_{membrane}$ is the internal resistance of nanochannels membrane, and $R_{solution}$ is the solution resistance. Considering that the ionic current measured is close to 0, we can reasonably neglect the contribution of iR drop. E_{redox} arises from the dependence of AgCl/Ag electrode potential on the activity of Cl^- and temperature,

$$E_{redox} = E_{cis} - E_{trans} = \frac{R}{F}(T_{trans} ln a_{trans} - T_{cis} ln a_{cis}) \tag{14}$$

Combining Supplementary equations **12, 13, and 14,** we obtain the following equation,

$$V_{oc} = -(\phi_{diff} + E_{redox}) = -2t_{+}\frac{R}{F}(T_{trans}lna_{trans} - T_{cis}lna_{cis}) \quad (15)$$

In this work, we studied the thermoelectric response for three cases, in the absence and presence of activity gradient across nanochannels. In the first case, there is no activity gradient across nanochannels (namely $a_{cis} = a_{trans} = a$). Both ϕ_{diff} and V_{oc} are equal to zero at the initial state ($T_{cis} = T_{trans}$). Upon changing the temperature of one solution (the temperature of another solution remained unchanged), they will vary and the magnitudes can be derived from Supplementary equations **12** and **15,** respectively,

$$\Delta\phi_{diff}(T) = (2t_{+} - 1)\frac{R}{F}\Delta T lna \quad (16)$$

$$\Delta V_{oc}(T) = -2t_{+}\frac{R}{F}\Delta T lna \quad (17)$$

where ΔT ($\Delta T = T_{trans} - T_{cis}$) is the magnitude of temperature change, namely the immediate temperature difference between two solutions separated by nanochannels.

In the second case, when the temperature of solution in *cis* chamber was changed (the temperature of solution in *trans* chamber remained unchanged), both ϕ_{diff} and V_{oc} will change in the same way.

In the third case (namely $a_{cis} \neq a_{trans}$), there exist nonzero ϕ_{diff} and V_{oc} at the initial state. If assuming the initial temperature of two solutions is T_0, they are associated with the activity gradient and expressed as,

$$\phi_{diff}(0) = (2t_{+} - 1)\frac{R}{F}(T_0 lna_{trans} - T_0 lna_{cis}) \quad (18)$$

$$V_{oc}(0) = -2t_{+}\frac{R}{F}(T_0 lna_{trans} - T_0 lna_{cis}) \quad (19)$$

In the similar way, when the temperature of solution in *trans* chamber was changed (the temperature of solution in *cis* chamber remained unchanged), both ϕ_{diff} and V_{oc} will change. Their net variations relative to the initial state are dependent on both temperature and activity gradients and can be expressed as,

$$\Delta\phi_{diff}(a,T) = \phi_{diff}(a,T) - \phi_{diff}(0) = (2t_{+} - 1)\frac{R}{F}\Delta T lna_{trans} \quad (20)$$

$$\Delta V_{oc}(a,T) = V_{oc}(a,T) - V_{oc}(0) = -2t_{+}\frac{R}{F}\Delta T lna_{trans} \quad (21)$$

Where a_{trans} is the electrolyte activity in the solution where the temperature changes.

Molecular Dynamics Simulation

For the simulation of the ion permeation through TpTag-COF, three layers of 3.78 nm × 3.27 nm TpTag-COF (4x4x3) were sandwiched by two solutions along the z-direction. One contains 2000 water molecules, 200 K^+ ions, and 200 Cl^- ions, and the other contains 2000 water molecules. A periodic boundary condition was applied to the x-y direction, and the length of the z-axis of the simulation box was 12.0 nm. TpTag-COF was optimized by the Universal Force Field (UFF) force field with the charge equilibrium (QEq) method[1]. The Simple Point Charge (SPC) model was used to describe water molecules[2]. The Optimized Potentials for Liquid Simulations All Atom (OPLS-AA) model was used to describe K^+ ions and Cl^- ions[3]. All MD simulations were performed by the package of Large-scale Atomic/Molecular Massively Parallel Simulator (LAMMPS) and visualized by OVITO software. The atoms of TpTag-COF were frozen during the simulation. The system was progressed to reach the energy minimization using the steepest descent approach before a 1 ns NVT simulation was performed (NVT: constant particle number, volume, and temperature). The initial velocities of K^+, Cl^-, and H_2O were assigned based on the Maxwell−Boltzmann distribution at 300 K and maintained by the Nose-Hoover thermostat. The simulation was run with a time step of 1 fs.

Supplementary Table 1|The detailed thermosensation sensitivity and t_+ values of TpTag-COF/PAN in the presence of various symmetric KCl aqueous solutions shown in Figure 4c.

Concentration (mM)	0.5	1	5	10	50	100
Sensitivity (mV K^{-1})[a]	1.27	1.19	0.89	0.79	0.54	0.40
t_+[a]	0.970	0.995	0.952	0.967	0.956	0.908

[a] average of 3 different batch experiments

Supplementary Table 2| The dependence of Debye screening length on the concentrations of KCl solution.

Concentration (mM)	1000	100	10	1	0.1
Debye length (nm)[a]	0.3	0.96	3.03	9.59	30.32

[a]The Debye length is defined as

$$\lambda_D = (\frac{\varepsilon_r \varepsilon_0 k_B T}{2 N_A e^2 I'})^{1/2}$$

where ε_r and ε_0 are the vacuum and relative permittivity, respectively, k_B is the Boltzmann constant, T is the absolute temperature, e is the elementary charge, N_A is the Avogadro number, and I is the ionic strength of the solution.

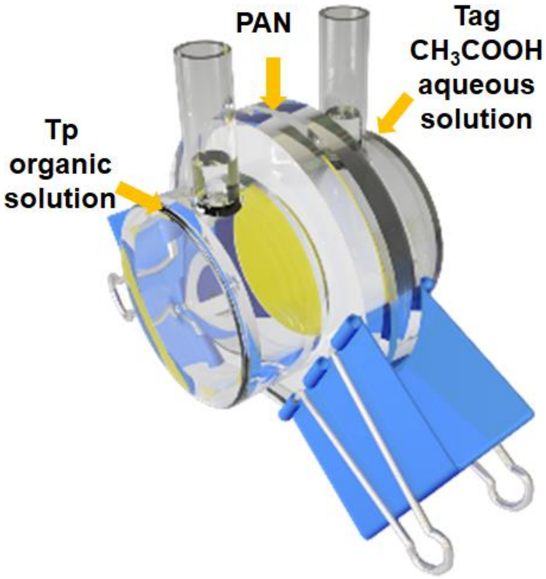

Supplementary Figure 1| Schematic illustration of the set-up used for the fabrication of TpTag-COF/PAN *via* interface polymerization.

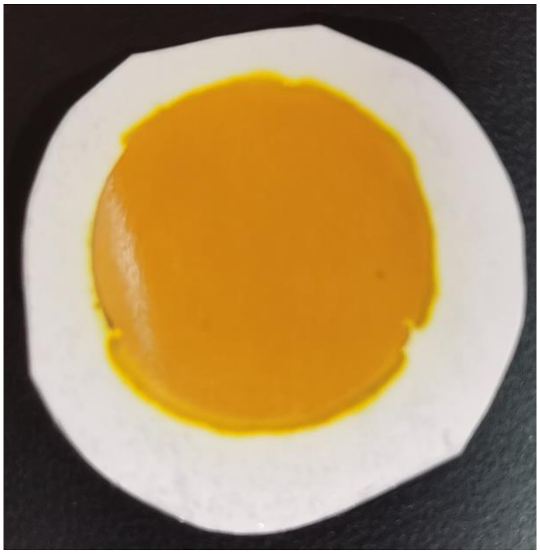

Supplementary Figure 2| Digital photos of TpTag-COF/PAN.

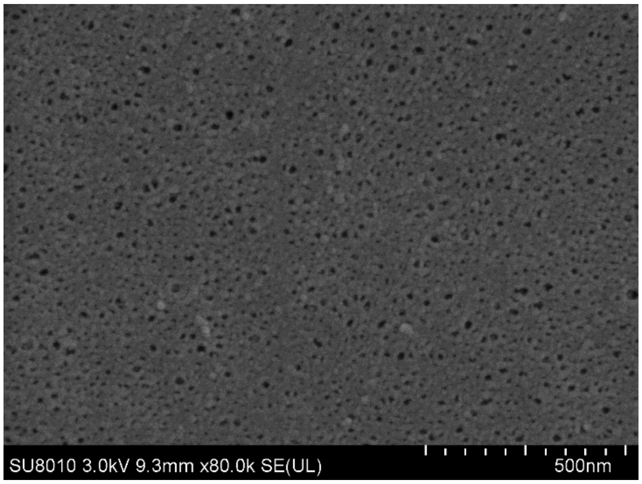

Supplementary Figure 3| Top-view SEM image of PAN.

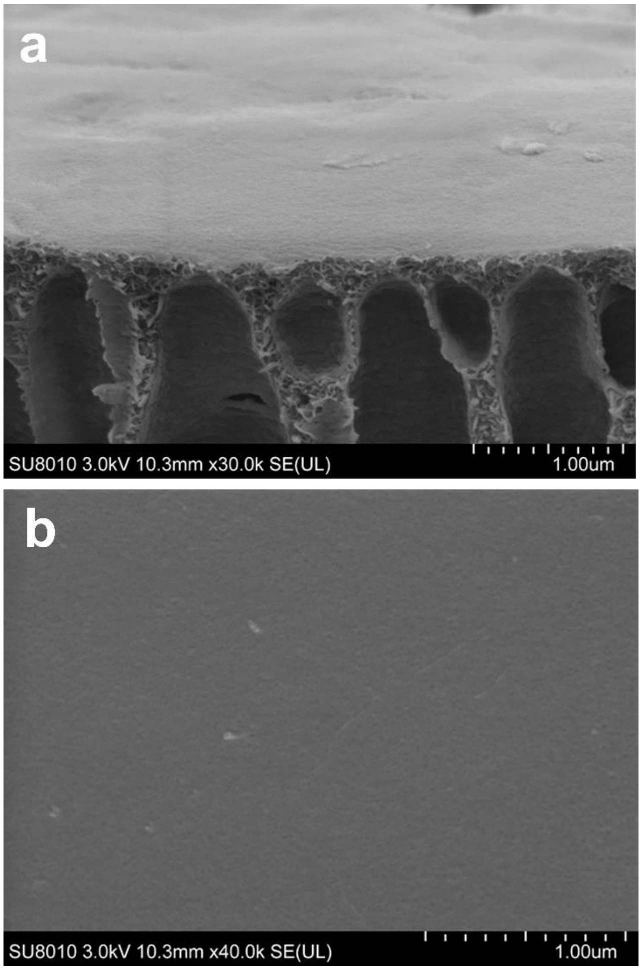

Supplementary Figure 4| SEM image. (a) cross-section-view and (b) Top-view SEM images of TpTag-COF/PAN.

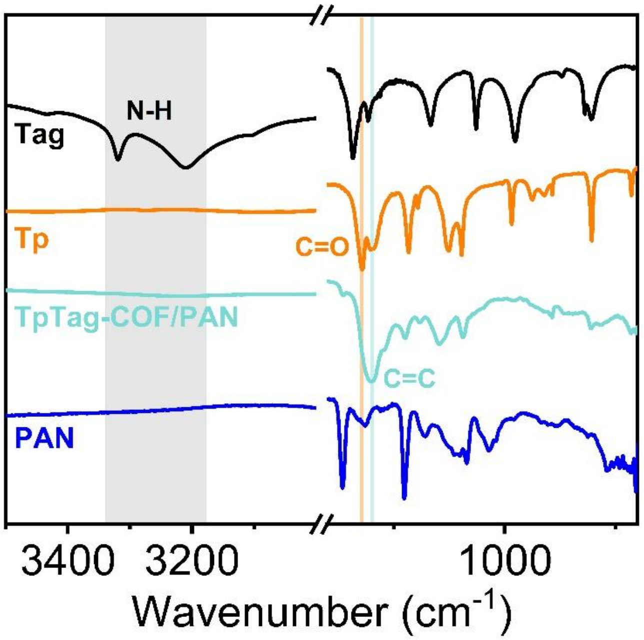

Supplementary Figure 5| IR spectra.

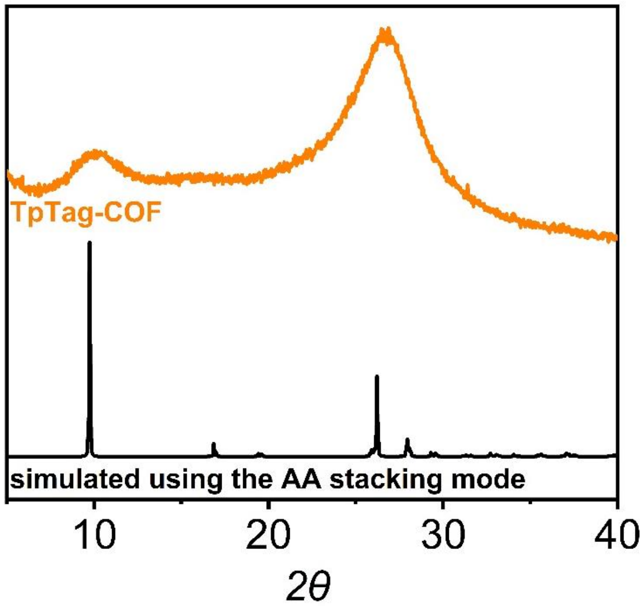

Supplementary Figure 6| Simulated and experimental PXRD patterns of TpTag-COF.

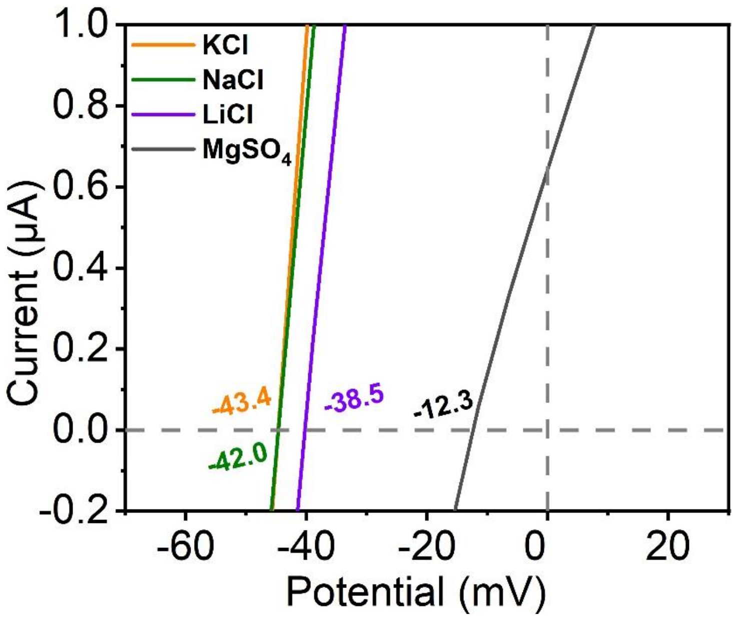

Supplementary Figure 7| Permselectivity evaluation. I−V plots of various electrolyte solutions with a concentration gradient of 100 mM and 10 mM separated by TpTag-COF/PAN.

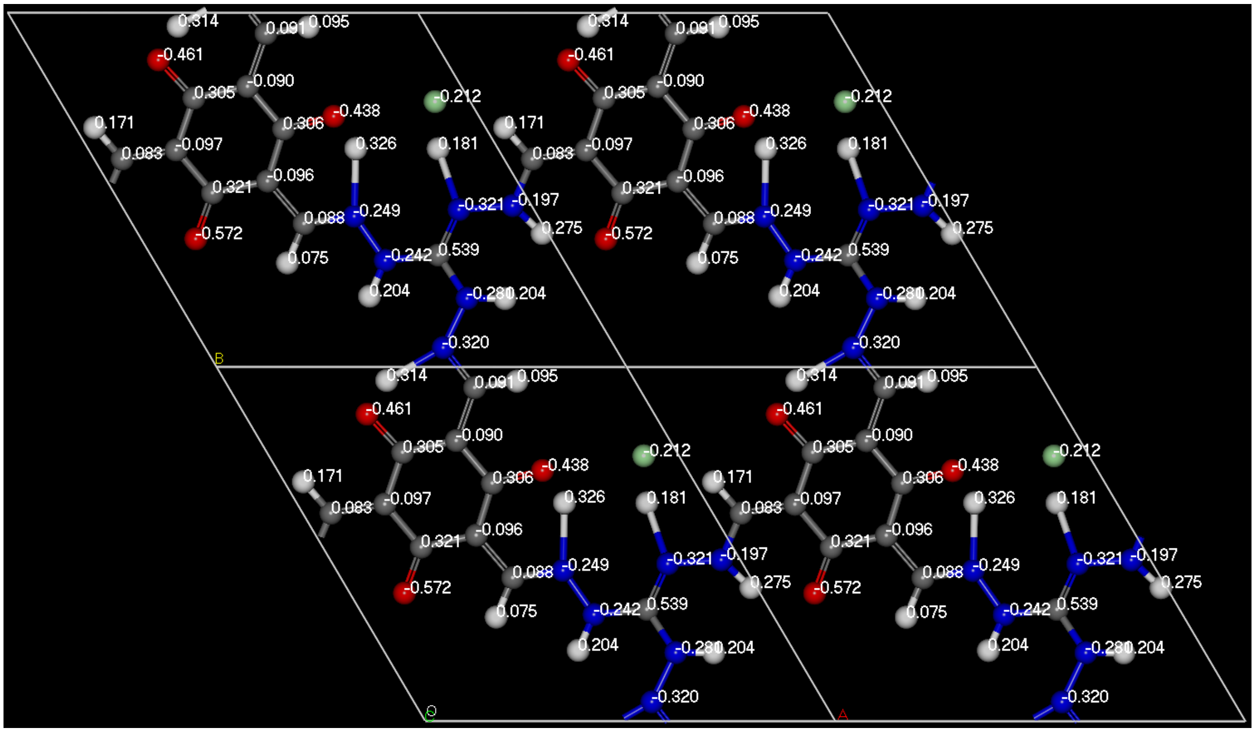

Supplementary Figure 8| The distributions of electrostatic potential of TpTag-COF/PAN, with the values labeled by white text.

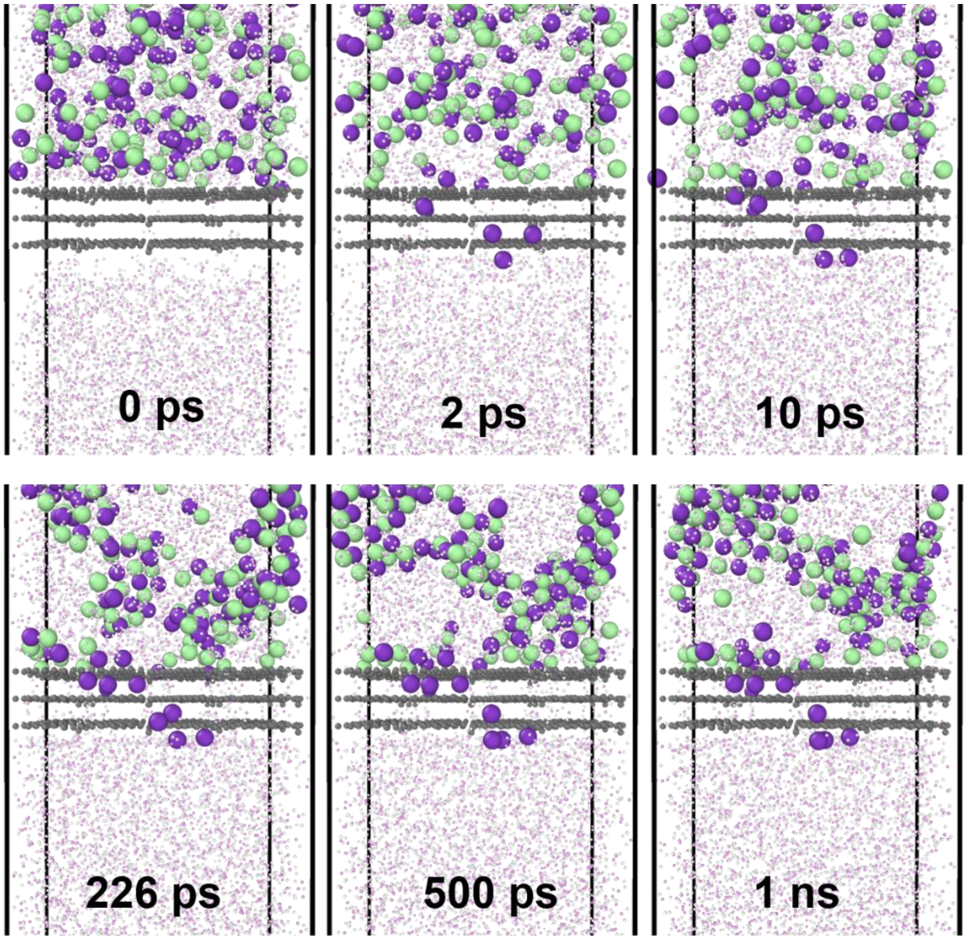

Supplementary Figure 9 | MD simulation. The schematic diagram of ion transport behavior through TpTag-COF subnanochannels at different intervals. The MD simulation revealed that TpTag-COF/PAN shows a higher K^+ ion transport activity than Cl^- ion (purple, K; green, Cl; red, O; white, H; gray, COF layers).

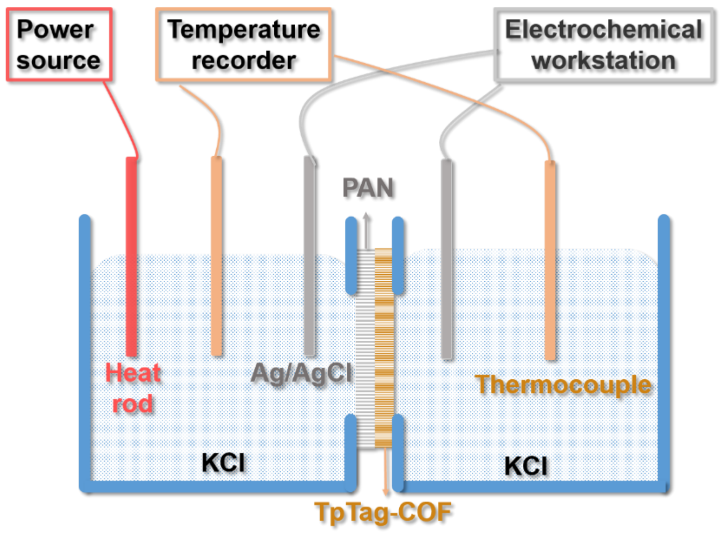

Supplementary Figure 10 | Schematic illustration of the experimental setup used for investigating the thermoelectric response of TpTag-COF/PAN.

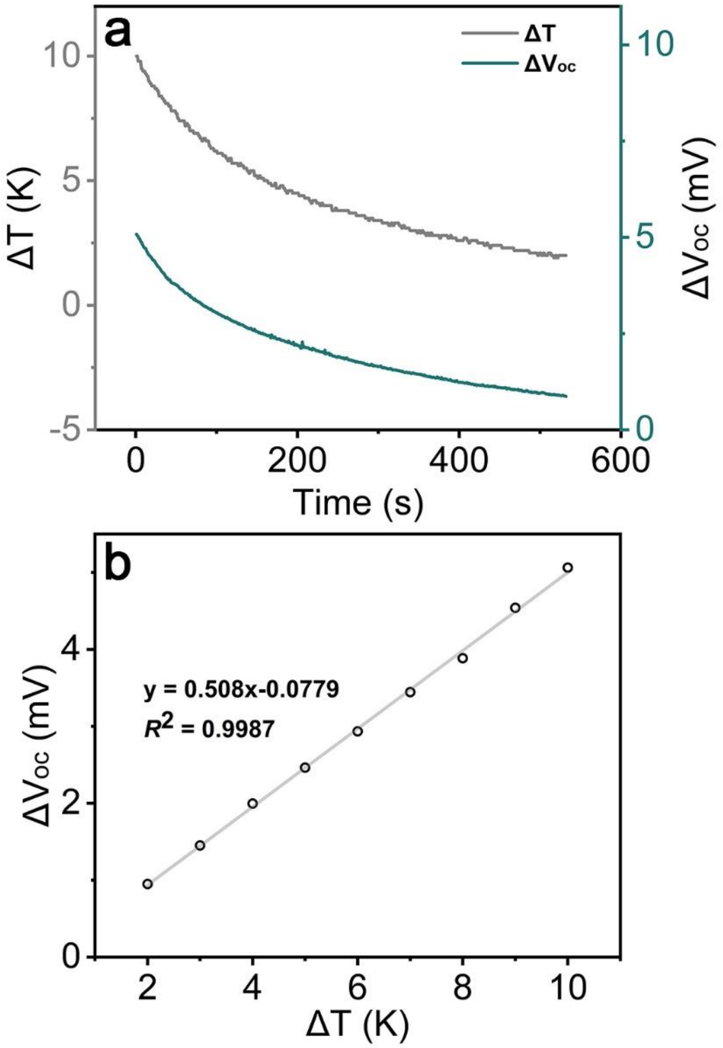

Supplementary Figure 11 | Thermosensation performance evaluation of PAN. (a) The synchronous time evolution ΔV_{oc} in response to the solution temperature change with the initial temperatures of 25 and 35 °C, respectively. (b) The linear fits of ΔV_{oc} against ΔT according to Equation 2 (main text). The corresponding thermosensation sensitivity and t_+ values derived from Equation 2 were estimated to be 0.51 mV K^{-1} and 0.625, respectively.

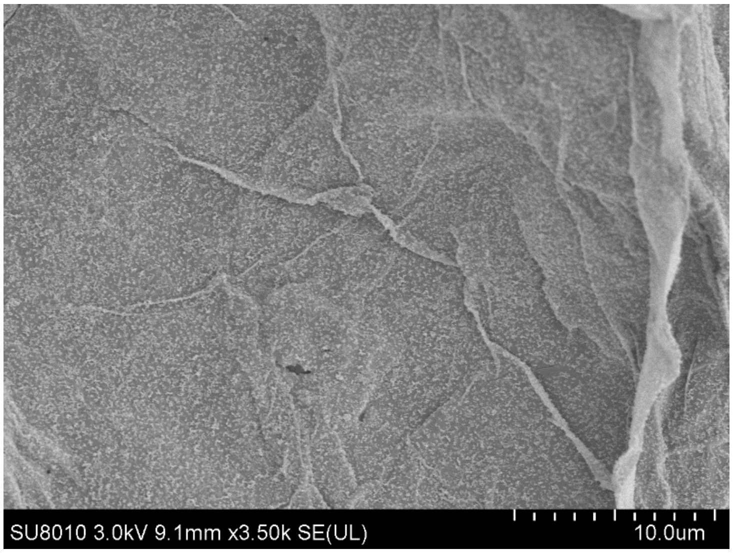

Supplementary Figure 12 | Top-view SEM images of BtTag/PAN.

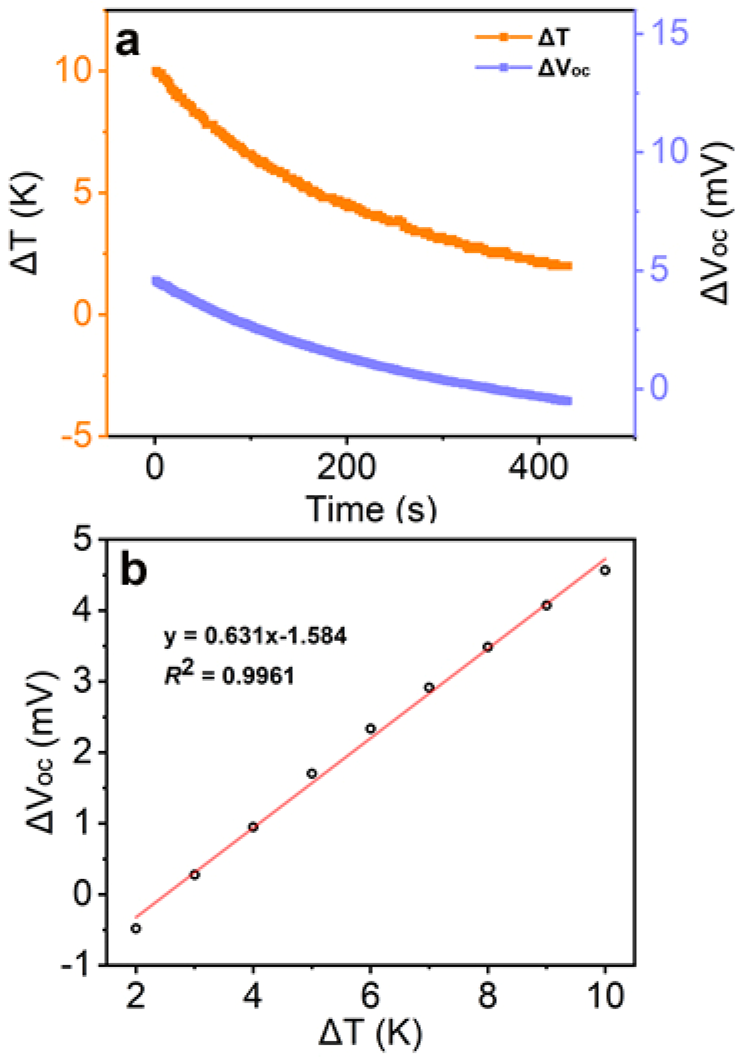

Supplementary Figure 13 | Thermosensation performance evaluation of BtTag/PAN. (a) The synchronous time evolution ΔV_{oc} in response to the solution temperature change with the initial temperatures of 25 and 35 °C, respectively. (b) The linear fits of ΔV_{oc} against ΔT according to Equation 2 (main text). The corresponding thermosensation sensitivity and t_+ values derived from Equation 2 were estimated to be 0.63 mV K^{-1} and 0.776, respectively.

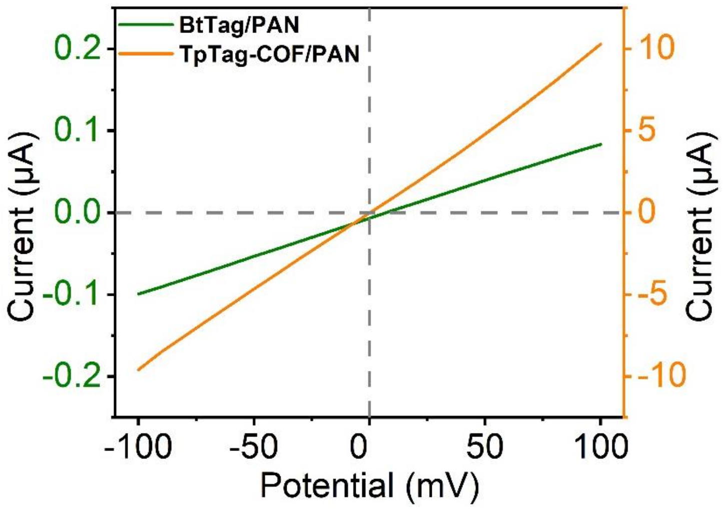

Supplementary Figure 14 | I–V curves of TpTag-COF/PAN and BtTag/PAN recorded in 10 mM KCl aqueous solution. The KCl conductance across TpTag-COF/PAN is over two orders of magnitude higher than that in BtTag/PAN (105/1).

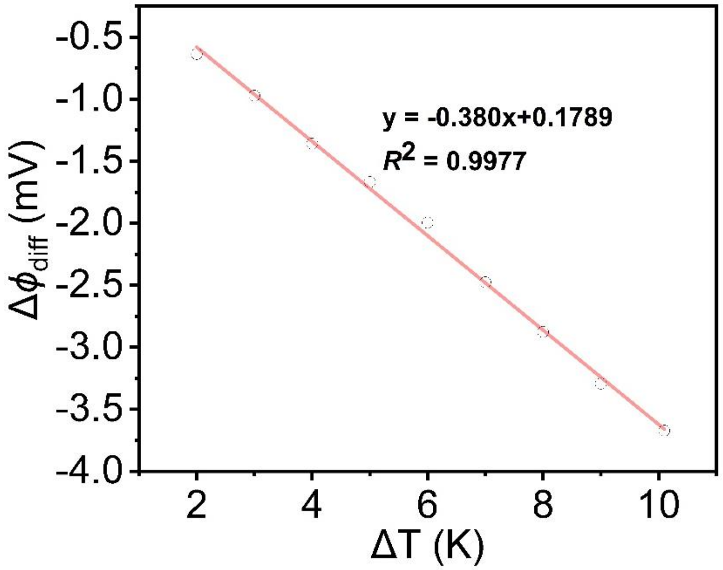

Supplementary Figure 15 | Dependence of $\Delta\phi_{diff}$ on ΔT. $\Delta\phi_{diff}$ values were estimated by subtraction of E_{redox} from ΔV_{oc}. The solid line corresponds to a linear fitting to the following equation:

$$\Delta\phi_{diff}\ (T) = -[\Delta E_{redox}(T) + \Delta V_{oc}(T)] = (2t_{+} - 1)\frac{R}{F}\Delta T ln a$$

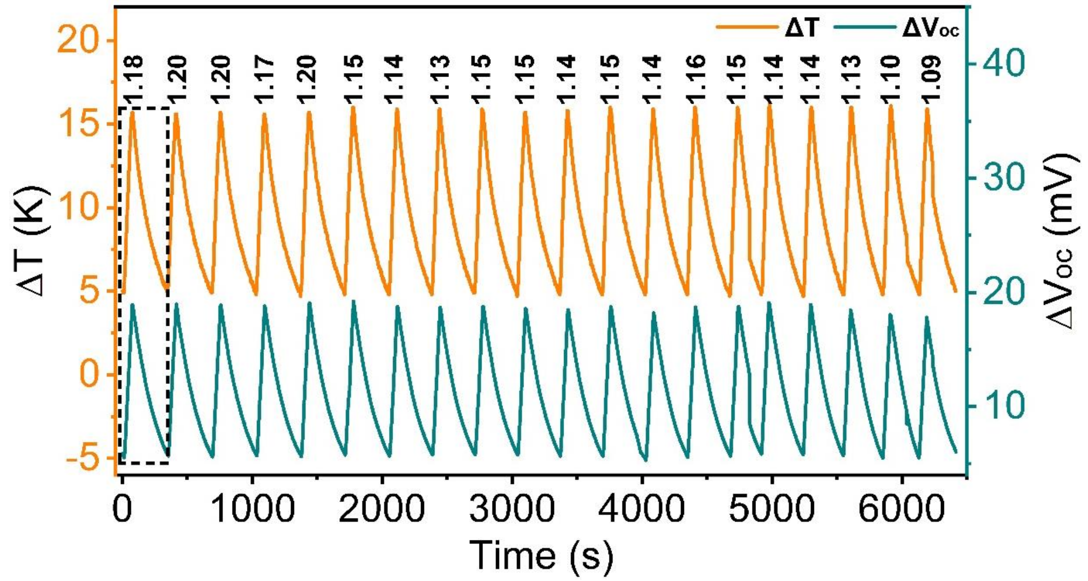

Supplementary Figure 16 | Stability evaluation. Continuous and synchronous time evolution curves of ΔV_{oc} (olive) and ΔT (orange) recorded with TpTag-COF/PAN membrane in1 mM KCl for 20 cycles. Inset numbers correspond to the thermosensation sensitivity of each cycle.

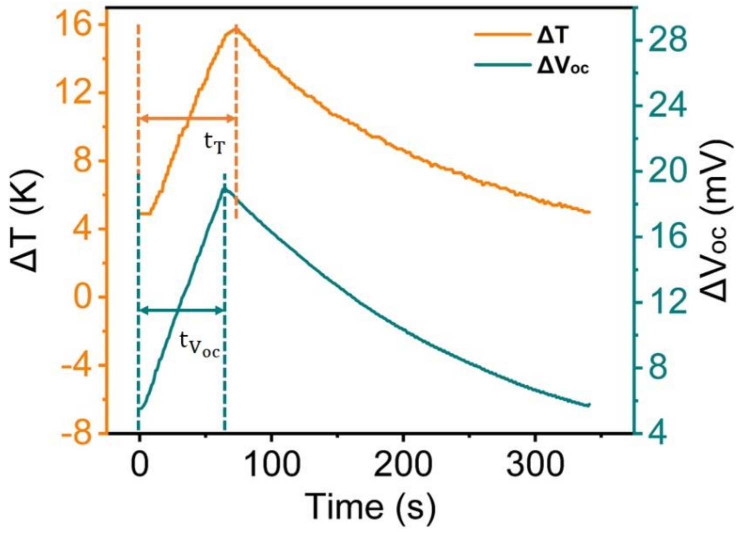

Supplementary Figure 17 | Thermosensation sensitivity evaluation. Enlarged section of black rectangle in Figure S9 and the representation of t_T and $t_{V_{oc}}$ shown in Equation 3 (main text).

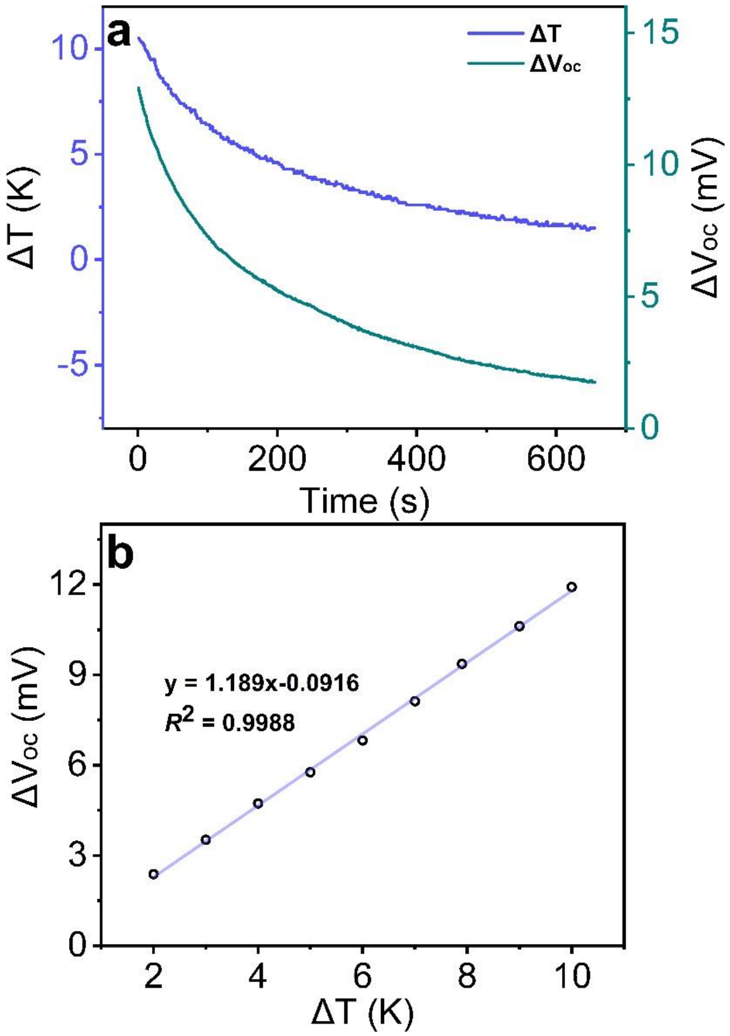

Supplementary Figure 18 | Thermosensation performance evaluation of TpTag-COF/PAN. (a) The synchronous time evolution ΔV_{oc} in response to the solution temperature change with the initial temperatures of 15 and 25 °C, respectively. (b) The linear fits of ΔV_{oc} against ΔT according to Equation 2 (main text). The corresponding thermosensation sensitivity and t_+ values derived from Equation 2 were estimated to be 1.19 mV K^{-1} and 0.994, respectively.

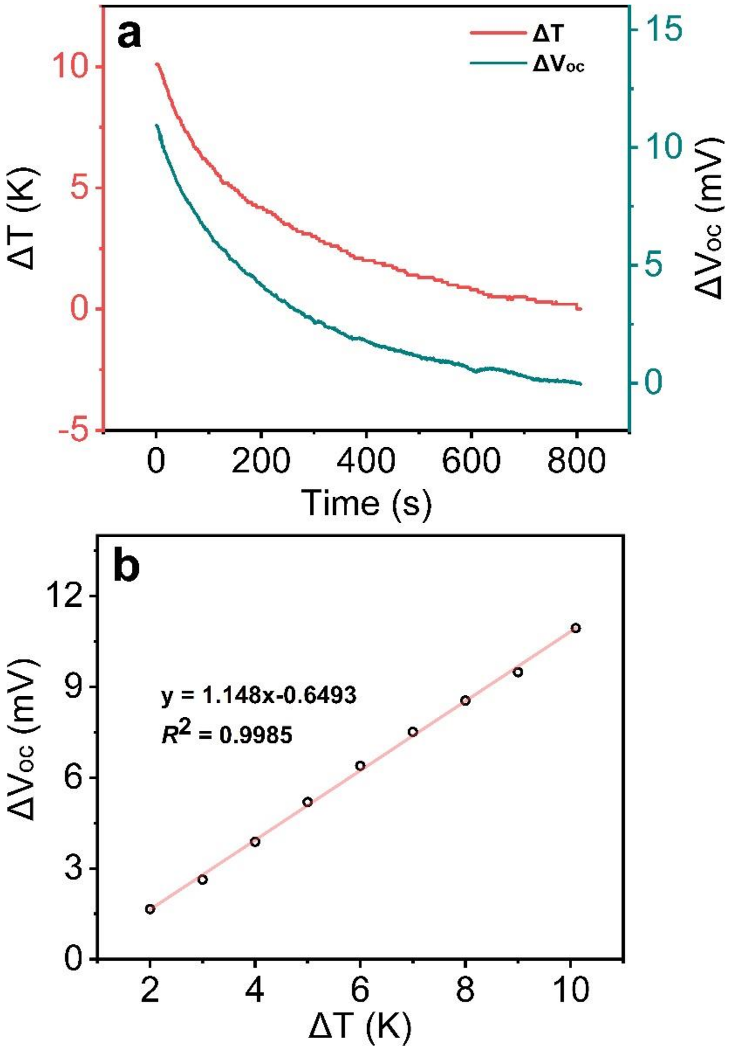

Supplementary Figure 19 | Thermosensation performance evaluation of TpTag-COF/PAN. (a) The synchronous time evolution ΔV_{oc} in response to the solution temperature change with the initial temperatures of 35 and 45 °C, respectively. (b) The linear fits of ΔV_{oc} against ΔT according to Equation 2 (main text). The corresponding thermosensation sensitivity and t_+ values derived from Equation 2 were estimated to be 1.15 mV K^{-1} and 0.961, respectively.

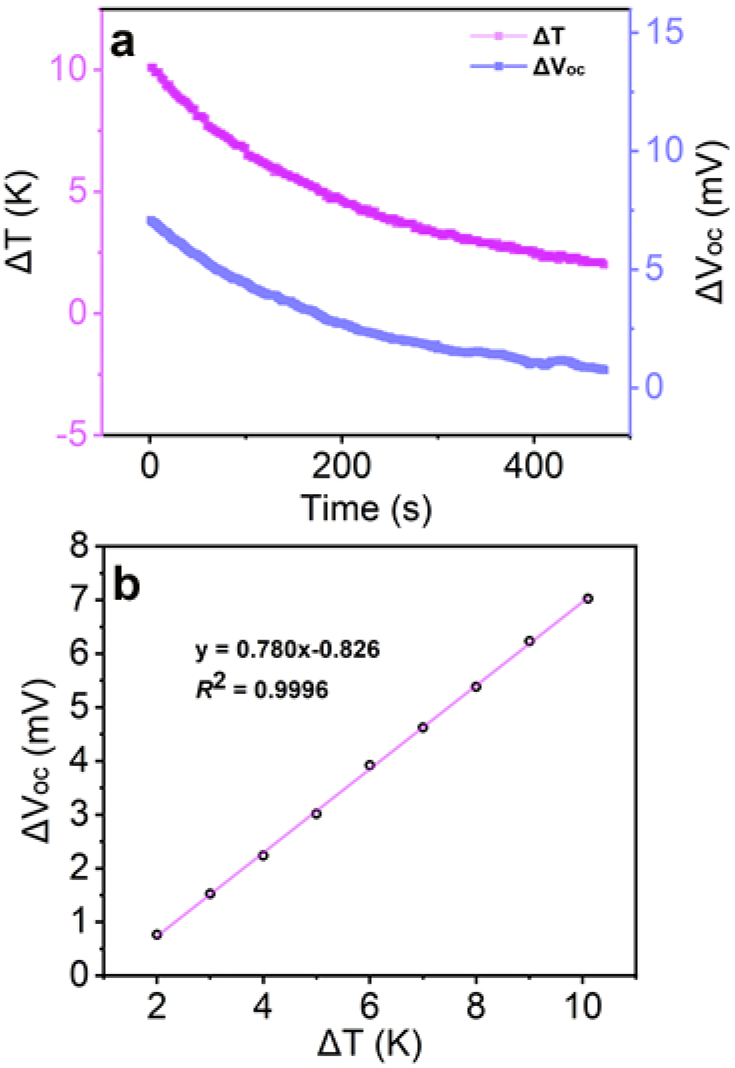

Supplementary Figure 20 | Thermosensation performance evaluation of TpTag-COF/PAN. (a) The synchronous time evolution ΔV_{oc} in response to the solution temperature change with the initial symmetric KCl solutions temperature (200 mM and 10 mM) of 25 °C. (b) The linear fits of ΔV_{oc} against ΔT according to Equation 2 (main text). The corresponding thermosensation sensitivity and t_+ values derived from Equation 2 were estimated to be 0.78 mV K^{-1} and 0.959, respectively.

Supplementary References

1. Kadantsev, E. S.; Boyd, P. G.; Daff, T. D. & Woo, T. K. Fast and accurate electrostatics in metal organic frameworks with a robust charge equilibration parameterization for high-throughput virtual screening of gas adsorption. *J. Phys. Chem. Lett.* **4**, 3056-3061 (2013).
2. Teleman, O.; Jönsson, B. & Engström, S. A molecular dynamics simulation of a water model with intramolecular degrees of freedom. *Mol. Phys.* **60**, 193-203 (1987).
3. Li, S.; Shi, Q.; Li, Y.; Yang, J.; Chang, T.- H.; Jiang, J. & Chen, P.-Y. Intercalation of metal Ions into $Ti_3C_2T_x$ MXene electrodes for high-areal-capacitance microsupercapacitors with neutral multivalent electrolytes. *Adv. Funct. Mater.* ***27***, 2003721 (2020).
